# MicroRNA-224 Induces G1/S Checkpoint Release in Liver Cancer

**DOI:** 10.3390/jcm4091713

**Published:** 2015-08-26

**Authors:** Fangmei An, Alexandru V. Olaru, Esteban Mezey, Qing Xie, Ling Li, Klaus B. Piontek, Florin M. Selaru

**Affiliations:** 1 Department of Gastroenterology, Wuxi People’s Hospital Affiliated to Nanjing Medical University, Wuxi, Jiangsu 214002, China; E-Mail: wdf8025@163.com; 2 Division of Gastroenterology and Hepatology, Department of Medicine, The Johns Hopkins Hospital, Baltimore, MD, 21205, USA; E-Mails: aolaru1@jhmi.edu (A.V.O.); emezey@jhmi.edu (E.M.); lli42@jhmi.edu (L.L.); kpiontek@jhmi.edu (K.B.P.); 3 Department of Infectious Diseases, Ruijin Hospital, Shanghai Jiao Tong University School of Medicine, Shanghai 200025, China; E-Mail: xieqingrjh@163.com; 4 The Sidney Kimmel Comprehensive Cancer Center, The Johns Hopkins Hospital, Baltimore, MD, 21287, USA

**Keywords:** cholangiocarcinoma, miR-224, cell cycle

## Abstract

Profound changes in microRNA (miR) expression levels are frequently found in liver cancers compared to the normal liver. In this study, we evaluate the expression of miR-224 in human HCC and CCA, as well as its downstream targets and affected pathways. We show that miR-224 is upregulated in a large cohort of human CCA, similar to its upregulation in human HCC. For the purpose of studying the roles of miR-224 in HCC and CCA, we enforced miR-224 expression in cells. mRNA arrays followed by Ingenuity Pathway Analysis (IPA)-identified putative molecules and pathways downstream of miR-224. Phenotypically, we report that enforced expression of miR-224 increases the growth rate of normal cholangiocytes, CCA cell lines, and HCC cell lines. In addition, we identified, in an unbiased fashion, that one of the major biologic processes affected by miR-224 is Gap1 (G1) to Synthesis (S) transition checkpoint release. We next identified p21, p15, and CCNE1 as downstream targets of miR-224 and confirmed the coordinated downregulation results in the increased phosphorylation of Retinoblastoma (Rb) with resulting G1/S checkpoint release. Our data suggest that miR-224 is a master regulator of cell cycle progression, and that its overexpression results in G1/S checkpoint release followed by accelerated cell growth.

## 1. Introduction

Liver cancers, including Hepatocellular Carcinoma (HCC) and Cholangiocarcinoma (CCA), display profound dysregulation in microRNA (miR) profiles compared to normal tissues [1]. While it is likely that some of these miRs are innocent bystanders or downstream of relevant, carcinogenesis-promoting molecular events, several reports implicated miR species at an etiologic level [2,3,4,5,6,7,8]. Although there is no information regarding its levels in human CCA, in a profiling study followed by a quantitative real-time polymerase chain reaction (qRT-PCR), miR-224 was found to be the most significantly overexpressed miR species in HCC [9]. In addition, miR-224 is the sole miR species to be upregulated in a variety of benign and malignant liver tumors, arising in a variety of predisposing liver conditions [10]. miR-224 was found to be upregulated in HCC (regardless of the etiology, including alcohol, hepatitis B, hepatitis C, hemochromatosis, idiopathic), hepatocellular adenomas (in a background of contraceptive use, beta-catenin mutations, and others), and focal nodular hyperplasia. Interestingly, the level of miR-224 paralleled the tumor growth potential, with normal levels in normal liver as well as in cirrhotic liver or liver infected with hepatitis B or C but statistically significantly higher levels in benign tumors and even higher levels in malignant tumors [10]. Furthermore, a different study found upregulation of miR-224 in dysplastic liver nodules, along with HCC [11]. Taken together, these data suggest that miR-224 might be involved at an early hepatic carcinogenetic stage and that its levels might be relevant to the liver cell growth potential. In the current study, we focused on the role of miR-224 in cell cycle regulation and subsequent cell growth in cancers arising from liver cells, including HCC and CCA. In addition, we set out to precisely determine pathways downstream of miR-224. We found that miR-224 is upregulated in human CCAs. In addition, we characterized the function of miR-224 based on mRNA targets that were experimentally downregulated rather than predicted based on web-based search engines. We found that miR-224 induces a G1/S checkpoint release through the downregulation of p21, p15, and CCNE1, resulting in increased phosphorylation of Retinoblastoma (Rb). Our findings were verified in CCA cells (HuCCT1 and CAK1 cells) and HCC cells (Huh7 and Hep3G cells) as well as in an SV-40-transformed normal cholangiocyte cell line (H69 cells). Combined, our findings implicate miR-224 as a salient regulator of cell growth in liver cancers, with potential therapeutic implications.

## 2. Experimental Section

### 2.1. Human Tissues

The human specimens were obtained from surgery performed at the Johns Hopkins Hospital (JHH, Baltimore, MD, USA) and the Mayo Clinic (Rochester, MN, USA). Informed consent was obtained from all patients in accordance with approved Johns Hopkins University and Mayo Clinic Institutional Review Board (IRB) protocols. Tissues were snap-frozen upon acquisition and stored in a −80 °C freezer until use. Total RNA was extracted from frozen tissues using Trizol Reagent (Cat# 15596018; Ambion, Carlsbad, CA, USA). 

### 2.2. Quantitative Real-Time RT-PCR (qRT-PCR)

qRT-PCR was used to evaluate the expression of miR-224. TaqMan miR assay kits (Applied Biosystems, Foster City, CA, USA) were used for miR-224 and normalized to RNU6B. Relative expression of target RNAs was calculated as 2^Ct_miR−Ct_RNU6B^. PCR reactions were carried out on the 7900 HT Fast Real-Time PCR System (Applied Biosystems, Carlsbad, CA, USA) in duplicate.

### 2.3. Cell Lines and Cell Culture

H69 cells (a gift from Dr. D. Jefferson, Tufts University, Boston, MA, USA) are normal human cholangiocytes transformed with SV-40. They were derived from a normal liver prior to liver transplantation [12], maintained in Dulbecco’s Modified Eagle Media (DMEM)/Dulbecco’s Modified Eagle Medium/Nutrient Mixture F-12 Ham (DMEM/F12) at a ratio 3:1, supplemented with 10% fetal calf serum (FCS), 1000 U/mL penicillin/streptomycin (P/S), Adenine, insulin, epinephrine, T3-T, EGF, and Hydrocortisone. The human CCA cell lines CAK-1 and HuCCT1 are derived from an extrahepatic bile duct cancer and from an intrahepatic CCA, respectively. These cells were maintained in DMEM supplemented with 10% FCS, 1000 U/mL P/S. Human hepatocarcinoma cell lines Huh7 and Hep3B were maintained in Dulbecco’s Modified Eagle Media (DMEM) supplemented with 10% FCS, 1000 U/mL P/S. Human colorectal adenocarcinoma cells, HCT116 cells and HCT116 Dicer negative cells (HCT116(-)), a generous gift from Dr. Bert Vogelstein [13], were maintained in DMEM supplemented with 10% FCS, 1000 U/mL P/S, 2% sodium bicarbonate, 1% sodium pyruvate, and 1% MEM non-essential amino acids.

### 2.4. Transfection with miR-224 Mimic or Non-Specific Mimic

The synthesized miR-224 mimic (miR-224M, Cat# C-300676-05) and non-specific mimic (NSM, Cat# CN-001000-01-10) were purchased from Dharmacon (Lafayette, LA, USA). 50%~70% confluent cells were transfected with 20 nM of miR-224M or miR-224ZIn using Lipofectamine RNAi MAX (Cat# 13778-150; Invitrogen, Carlsbad, CA, USA). NSM or NSI were used as negative controls, respectively. RNA and proteins were harvested 48 and 72 h after transfection, respectively. 

### 2.5. Retroviral Vectors, Viral Supernatant Production, and Viral Transduction

MSCV-based bicistronic retroviral vectors MIEG3 [14] were used to express miR-224. The genomic DNA sequence of miR-224 was amplified using PCR primers digested by EcoRI (5′) and XhoI (3′) and cloned into the multiple cloning site of MIEG3. The primers for genomic miR-224 were: GGCGAATTCGAATTCCTCTTCTGCCAGCTA (forward) and GCGCTCGAGCGAGCGGCCGCCAGTGTGAT (reverse). The expression of miR-224 was linked with expression of enhanced green fluorescence protein (eGFP) via internal ribosome entry site 2 (IRES2). The plasmid DNA was used to generate viral supernatant from 293-T cells as previously described [15]. Briefly, 293-T cells were grown to 70% confluence in a T75 tissue culture-treated flask (Corning, Inc., Corning, NY, USA). Eight micrograms (μg) of plasmid DNA of miR-224 together with 10 μg MLV gag-pol plasmid and 3 μg Vesicular stomatitis virus envelope glycoprotein (VSV-G) plasmid were co-transfected using Lipofectamine 2000 (Cat# 11668-019; Invitrogen, Carlsbad, CA, USA). Eight milliliters (mL) of viral supernatant were collected every 24 h and stored at −80 °C until used. Then, 3 × 10^5^ H69, HuCCT1, Huh7, and HCT116 cells were cultured with 2 mL of viral supernatant containing 8 mg/mL of hexadimethrine bromide (Polybrene, Cat# 107689; Sigma-Aldrich, Milwaukee, WI, USA), respectively. After 6–8 h, the viral supernatant was discarded and fresh DMEM media was added. Two days after transduction, cells were harvested and sorted for eGFP expression using a fluorescence-activated cell sorter (FACSVantage SE DiVa, Becton Dickinson, San Jose, CA, USA). Then, we got the cells stably expressing miR-224 (MIEG3-224V), and the empty MIEG3 virus was used as control (MIEG3-EV). 

### 2.6. Cell Growth Assay

Ten thousand cells were plated in 24-well plates and transfected 24 h later (Day 0) and counted every other day using a hemocytometer and an inverted-light microscope. 

### 2.7. cDNA Microarrays and Filtering Genes

The Illumina cDNA microarray platform in the Johns Hopkins genomics facility was used for cDNA microarrays. HCT116 Dicer negative cells were treated with miR-224M or NSM, and 72 h later, the RNA was extracted. Candidate genes were filtered as follows: Genes with expression in HCT116 Dicer negative cells under 3000 units were eliminated from analysis due to low expression. Genes that demonstrated less than 20% decrease upon stimulation with miR-224 were eliminated. These genes were input into Ingenuity Pathway Analysis (IPA) to identify the pathways in which they are involved.

### 2.8. Cell Cycle Analysis by Flow Cytometry

Flow cytometry analysis of DNA content was performed to assess cell cycle phase distribution. HuCCT1 cells with MIEG3-EV and MIEG3-224V infection were cultured in DMEM media without FCS (serum starved) for 72 h and maintained in growth media for another 30 h; then the cells were harvested and incubated with Propidium iodide (PI) staining buffer (PBS 0.1 mg/mL PI, 0.6% NP40, 2 mg/mL RNase A for 30 min on ice (Roche Diagnostics). The DNA content was analyzed using FACSCalibur (BD Biosciences, San Jose, CA, USA) and Cell Quest software (BD Biosciences) for histogram analysis. Similarly, H69 cells were either transfected and analyzed 72 h later, or transduced with MIEG3-EV and MIEG3-224V, respectively, and analyzed at baseline, after Doxorubicin treatment (0.1 ug/mL for 24 h), and after serum starvation, respectively.

### 2.9. Luciferase Reporter Assay

*PGL4 luciferase plasmid construction:* According to TargetScan [16], miR-224 has a binding site in p21 3′-untranslated region (3′-UTR) at the position of 1524–1530. A portion of the P21 3′UTR containing miR-224 predicted binding site (wild-type) was amplified using linker primers containing XbaI restriction sites. Primers for the wild-type were the following: 5′- GCTCAATAAATGATTCTTAGTGACTTTTCTAGA-3′ (forward) and 5′-GCGTCTAGAACAGG AACCAAGAACAAG-3′ (reverse). The amplicons were cut by XbaI and cloned into an XbaI site just downstream of the firefly luciferase structural gene in vector pGL4 (Cat# E6651; Promega, Madison, WI, USA). For the 3'UTR mutant, the miR-224 binding site was mutated by substituting the eight nucleotides of the miR-224 binding sites using the Gene Tailor site-directed mutagenesis system (Cat# 4500239; Invitrogen, Carlsbad, CA, US). Primers for the mutations were the following: 5′- GCTCAATAAATGATTCTTAGCAGCTTTTCTAGA-3′ (forward) and 5′-AAGAAGGAAAAAGAGGCTCCAAGAG-3′ (reverse). All plasmids (wild-type and mutant) were verified by sequencing. After sequence verification, we obtained plasmid clones containing correctly oriented inserts. Six thousand cells per well were seeded onto 96-well plates 24 h prior to transfection. Cells were transfected with miR-224M or the NSM. Then, 24 h after transfection, the cells were co-transfected with constructed wild-type or mutated pGL4 vector (firefly luciferase) and internal control pRL-CMV (Renilla luciferase, Cat# E2261; Promega, Madison, WI, USA) vector. Next, 48 h after plasmid vector transfection, the luciferase reporter assay was performed using a Dual-Luciferase Reporter Assay System (Cat# P1041; Promega, Madison, WI, USA). After 48 h, luminescence intensity was measured by Veritas Microplate Luminometer (Turner Biosystems, Madison, WI, USA), and the luminescence intensity of firefly luciferase was normalized to that of Renilla luciferase.

### 2.10. Western Blotting

Cells were lysed in Laemmli sample buffer (Cat#161-0737; Bio-Rad, Hercules, CA, USA) supplemented with a protease inhibitor (cOmplete, EDTA-free, Roche, Indianapolis, IN, USA). Protein concentration was measured using a Bicinchoninic Acid Assay (BCA) Protein Assay kit (Cat# 23227; Thermo Scientific, Rockford, IL, USA). Cell lysates (40–45 μg per lane) were electrophoresed on 10%–20% polyacrylamide gels (Cat# 456-1084; Bio-Rad, Hercules, CA, USA) and transferred to Immobilon-PSQ membranes (Millipore, Bedford, MA, USA). The membranes were blocked with Tris-buffered Saline (TBS) containing 5% skim milk and 0.1% Tween-20 (TBST), then incubated with the primary antibody. Antibody to p15 was purchased from Santa Cruz (SC613; Santa Cruz, CA, USA), antibody to p21 was purchased from Cell Signaling (2947, Cell Signaling, Danvers, MA, USA), antibody to CCNE1 was purchased from Santa Cruz (SC481, Santa Cruz, CA, USA), antibody to Phospho-Rb was purchased from Cell Signaling (3590, Cell Signaling, Boston, MA, USA). The membranes were incubated after TBST washing with HRP-conjugated anti-rabbit (A21109; Invitrogen, Frederick, MD, USA) secondary antibody, respectively, and analyzed using enhanced chemiluminescent Horseradish Peroxidase (HRP) Antibody Detect Reagent (Cat# E2400; Denville Scientific, Inc., Metuchen, NJ, USA).

### 2.11. Statistical Analysis

All data are presented as means ± Standard Deviation (SD). Student’s *t*-test and one-way ANOVA were performed for comparing continuous variables of two and three or more groups, respectively. Differences between group means with *p* values <0.05 were regarded as being statistically significant.

## 3. Results and Discussion

### 3.1. miR-224 is Upregulated in Human CCA vs. Normal Tissues

**Figure 1:**
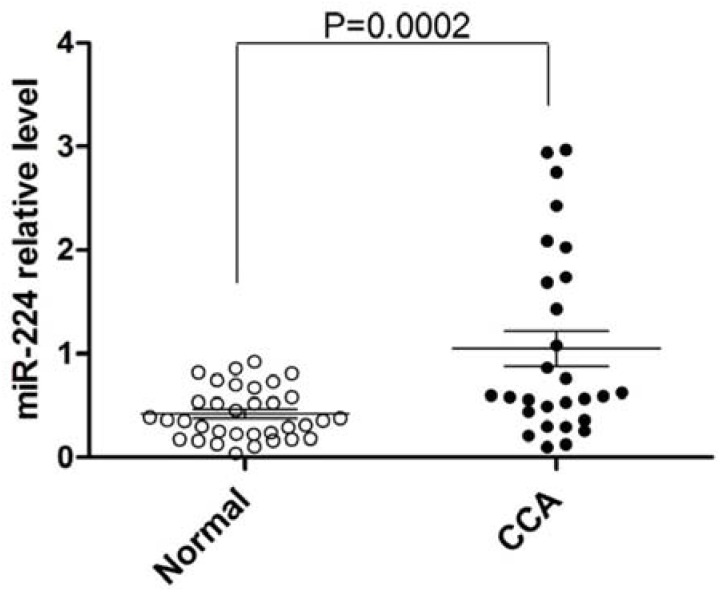
miR-224 is upregulated in CCA *vs.* normal biliary epithelium. The figure displays the mean and standard deviation of qRT-PCR-measured expression of miR-224 normalized to RNU6B for human CCA (filled circles) and normal liver tissues (open circles). Y-axis: qRT-PCR expression of miR-224 *vs.* RNU6B. Open circles indicate normal liver tissues and filled circles indicate CCA tissues.

We assayed the expression of miR-224 in a large cohort of 62 human specimens, including 28 CCAs and 34 normal liver tissues. The average expression of miR-224 *vs.* RNU6B in CCA was higher than in normal tissues (*p*-value < 0.001, unpaired Student’s *t*-test, (Figure 1)). 

### 3.2. miR-224 Induces Cell Growth

We started exploring the function of miR-224 by performing cell line transfections. However, we noted that following transfections, miR-224 was upregulated to levels that were un-physiologically high. To accomplish a lower, more physiological level of miR-224 upregulation than in transfection experiments, we inserted the genomic locus of miR-224 in a retrovirus, MSCV-IRES-Enhanced-GFP-3 (MIEG3). We then infected HuCCT1, H69, Huh7, and HCT116 Dicer (-) with MIEG3-EV (empty vector, EV) and MIEG3-miR-224V (miR-224V), respectively, and determined the level of miR-224 upregulation. Compared to cells infected with MIEG3 alone (EV), cells infected with miR-224V displayed a 3.4- and 3.6-fold upregulation of miR-224 in HuCCT1 and Huh7 cells, respectively (Supplementary Figure 1A,B). 

**Figure 2:**
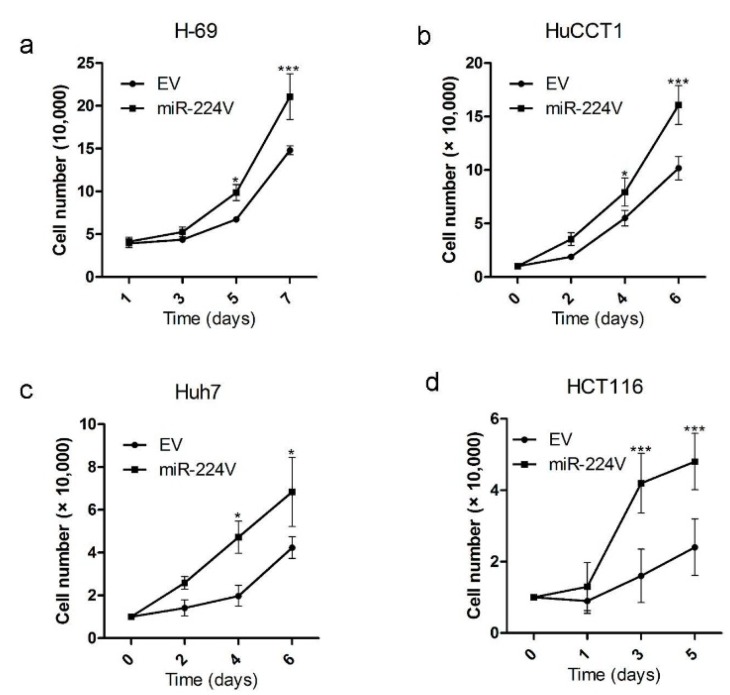
Cells display increased growth upon modest miR-224 upregulation through infection with MIEG3-miR-224.H-69 (**a**), HuCCT1 (**b**), Huh7 (**c**), and HCT116 (-) (**d**); cells were counted at different days after plating of MIEG3-miR-224 (miR-224V) or MIEG3-EV (EV) cells, respectively. Cell counts × 10^4^ of cells at days indicated infected with miR-224V (squares) or the EV (circles). For every treatment and every time point, the data presented are the average of five independently counted wells. Mean ± SD, * *p* < 0.05, *** *p* < 0.001.

This level of upregulation is approximately the same as in human CCA specimens. As shown in Figure 2, miR-224 induces increased growth in H69, HuCCT1, and Huh7, as well as in HCT116(-) cells. 

### 3.3. miR-224 Functions and Signaling Pathways Analysis

To obtain a mechanistic view into the effects of miR-224 in cancer cells, and since miR-induced destabilization of mRNA is the main reason for decreased protein levels [17], we stimulated HCT116 Dicer (-) with a miR-224 mimic and performed cDNA microarray analysis to quantify changes in mRNA levels. Cells that lack Dicer completely are not viable, but these HCT116 hypomorphic Dicer cells exhibit reduced mature miRNA levels [13]. This increases the expression level changes caused by treatment with a miR mimic at least two-fold [18] and, hence, we chose HCT Dicer (-) cells to obtain better sensitivity of miR-224-targeted mRNAs. The list of genes identified to be downregulated upon miR-224 stimulation was then filtered and input into Ingenuity Pathway Analysis (IPA, Ingenuity Systems Incorporated, Redwood City, CA, USA), with the purpose of identifying general mechanisms of miR function. It is of note that this analysis was performed on mRNA species that are reported to be downregulated by miR-224 on the cDNA arrays, irrespective of the presence of the binding site in the 3′UTR or *in silico* search engine prediction. IPA reported that the top one disease regulated by miR-224 was “Cancer” and the top three molecular and cellular functions of the genes regulated by miR-224 were “Gene Expression”, “Cell Cycle”, and “Cell Death”, and the top two ranked canonical pathways of the genes regulated by miR-224 were reported to be “Regulation of Actin-based Motility by Rho” and “Cell Cycle Regulation by BTG Family Proteins” (Table 1). Furthermore, according to the cDNA array, after the network between genes and diseases regulated by miR-224 was constructed, it demonstrated that many genes regulated by miR-224 were involved in cancer and liver diseases. From this point on, we focused on the cell cycle effects of miR-224.

**Table 1:** Prediction of functions and signal pathways. Based on the mRNA array data, Ingenuity Pathway Analysis (IPA) was performed. The IPA reports places “Cancer” as the top one disease regulated by miR-224, while the top three molecular and cellular functions associated with the list of genes regulated by miR-224 were listed as “Gene Expression”, “Cell Cycle”, and “Cell Death”, and the top two ranked canonical pathways of the genes regulated by miR-224 were reported to be “Regulation of Actin-based Motility by Rho” and “Cell Cycle Regulation by BTG Family Proteins”. Molecules Number indicates the number of molecules that were regulated by miR-224 involved in functions and signal pathways. BTG: B-cell translocation gene, VDR: Vitamin D receptor, RXR: Retinoid X receptor, AST: aspartate aminotransferase.

**Diseases and Disorders**		
**Name**	***p*-Value**	**Molecules Number**
Cancer	1.24E-05–1.35E--02	188
Immunological Disease	2.15E-05–1.01E--02	34
Neurological Disease	1.05E-04–1.33E--02	179
Dermatological Diseases and Conditions	1.11E-04–1.27E--02	76
Genetic Disorder	1.39E-45–1.27E--02	113
**Molecular and Cellular Functions**		
**Name**	***p*-Value**	**Molecules Number**
Gene Expression	5.20E-07–1.15E--02	137
Cell Cycle	8.78E-07–1.36E--02	90
Cell Death	2.71E-06–1.12E--02	129
Cellular Growth and Proliferation	9.88E-06–1.40E--02	162
Small Molecule Biochemistry	5.00E-05–1.18E--02	61
**Physiological System Development and Function**		
**Name**	***p*-Value**	**Molecules Number**
Connective Tissue Development and Function	1.28E-04–1.26E--02	58
Embryonic Development	2.22E-04–9.57E--03	33
Hematological System Development and Function	2.32E-04–1.38E--03	72
Hematopoiesis	2.32E-04–1.38E--03	48
**Top Canonical Pathways**		
**Name**	***p*-Value**	**Ratio**
Regulation of Actin-based Motility by Rho	7.22E-05	1/19(0.121)
Cell Cycle Regulation by BTG Family Proteins	1.01E-04	7/36(0.197)
Pancreatic Adenocarcinoma Signaling	5.76E-04	11/119(0.092)
Crosstalk between Dendritic Cells and Natural Killer Cells	5.91E-04	10/96(0.104)
Type 1 Diabetes Mellitus Signaling	7.29E-04	11/121(0.091)
**Top Tox Lists**		
**Name**	***p*-Value**	**Ratio**
Renal Necrosis/Cell Death	1.04E-06	28/314(0.089)
Oxidative Stress	2.17E-03	7/57(0.123)
Cell Cycle:G1/S Checkpoint Regulation	9.47E-03	6/57(0.105)
Aryl Hydrocarbon Receptor Signaling	1.22E-02	11/157(0.07)
VDR/RXR Activation	1.23E-02	7/78(0.09)
**Clinical Chemistry and Hematology**		
**Name**	***p*-Value**	**Molecules Number**
Increased Levels of AST	1.40E-02–1.40E--02	2
Increased Levels of Albumin	3.20E-02–3.20E--02	1
Increased Levels of Alkaline Phosphatase	6.29E-02–3.79E--02	3
Increased Levels of Potassium	6.29E-02–6.29E--02	1
Decreased Levels of Albumin	1.77E-01–1.77E--01	1
**Hepatotoxicity**		
**Name**	***p*-Value**	**Molecules Number**
Liver Damage	5.86E-03–1.41E--01	5
Liver Necrosis/Cell Death	1.15E-02–3.45E--01	12
Liver Cholestasis	2.51E-02–1.19E--01	7
Biliary Hyperplasia	3.20E-02–3.20E--01	1
Liver Degradation	3.20E-02–3.20E--02	1

### 3.4. miR-224 Induces G1/S Checkpoint Release in Normal and Cancer Cells

**Figure 3:**
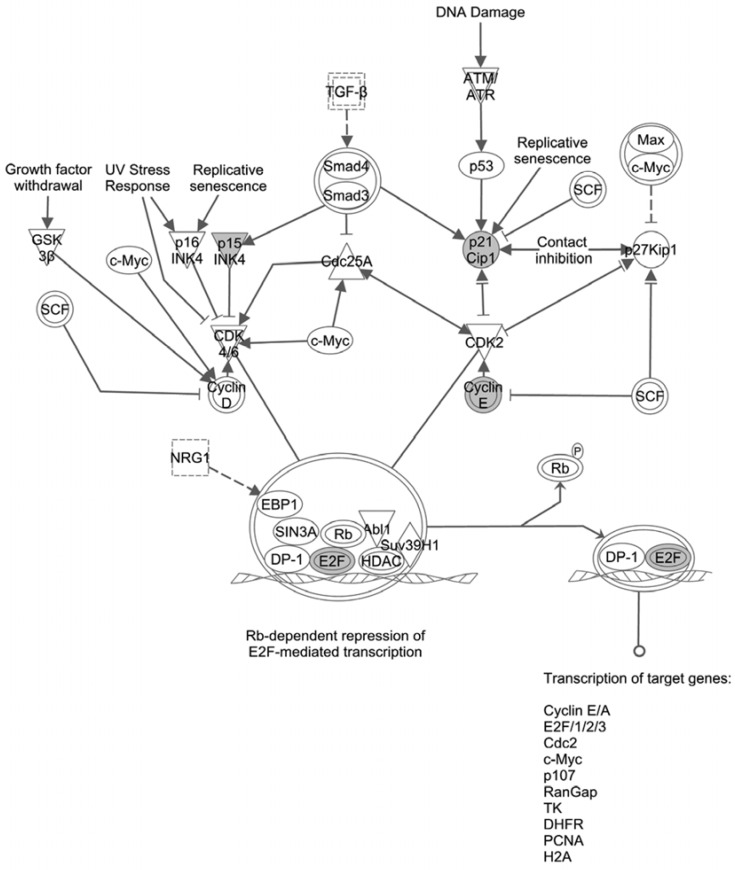
Genes with altered expression upon miR-224 stimulation are involved in the G1/S checkpoint. P15, p21, and CCNE1 were identified to be regulated by miR-224. They are involved in the final steps of G1/S checkpoint regulation. Decreasing levels of these molecules would result in increased phosphorylated Rb (p-Rb), with the end result being the release from the G1/S checkpoint.

To study the molecular mechanisms responsible for the miR-224-induced cell cycle dysfunction, we queried IPA in regards to genes impacted by miR-224 that are also involved in the G1/S checkpoint. As Figure 3 shows, based on cDNA microarray data, miR-224 appears to regulate several molecules involved in the G1/S checkpoint. The mRNA levels of p15 (INK4), p21(Cip1), and Cyclin E1 (CCNE1) decreased following miR-224 stimulation. 

**Figure 4:**
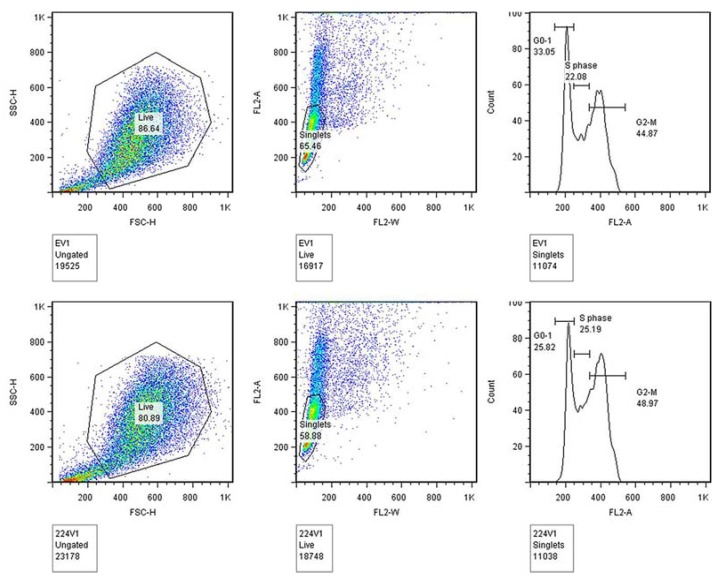
HuCCT1 cells display G1 release upon reinforced miR-224 expression. Flow cytometric analysis of cell cycle via PI staining of HuCCT1 cells infected with MIEG3-miR-224 (224V, lower panels), or MIEG3 only (EV, upper panels). X-axis: DNA content as measured by PI incorporation; y-axis: cell counts for each phase of the cell cycle. The figure is representative of three experiments with three replicates per experiment.

To identify the precise effects of miR-224 on the cell cycle in CCAs, HuCCT1 cells were cultured in growth media without serum (serum-starved) for 72 h and then cultured with the growth media for another 30 h; then the cells were collected and cell cycle analysis was performed by PI staining. This experiment revealed a noticeable decrease in G0 to G1 phase and a corresponding increase in G2 to M phase in miR-224 overexpressed HuCCT1 cells (Figure 4).

### 3.5. miR-224 Regulates Multiple Proteins Involved in G1/S Checkpoint

**Figure 5:**
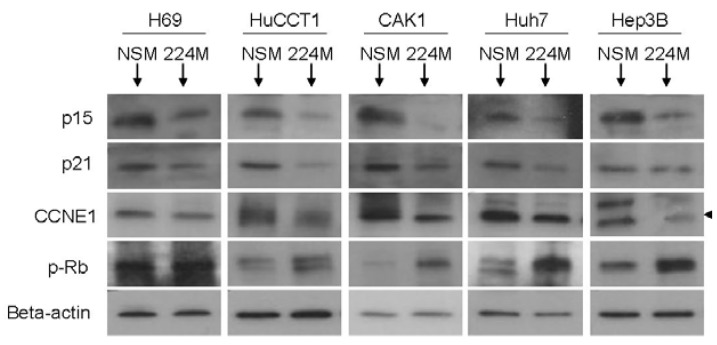
Protein expression of miR-224 target genes decrease upon miR-224 stimulation. Representative Western blots for p15, p21, CCNE1, and phospho-Rb are shown. The arrow indicates the predicted molecular size of CCNE1 of 53 kDa. Equal protein loading is shown by probing against beta-actin.

To verify whether miR-224 impacts the protein levels of these targets, H69, HuCCT1, CAK1, Huh7, and Hep3B cells, respectively, were treated with miR-224 mimic, and Western blotting was performed in these cell lines for the putative targets. As seen in Figure 5, the expression of miR-224 results in the decreased protein levels of p15, p21 and CCNE1. We then hypothesized that if the effects of miR-224 on these proteins are significant, the final step in the G1 to S transition checkpoint should also be affected. We therefore determined whether cells treated with miR-224 showed increased phosphorylation of Rb (p-Rb). In accordance with our hypothesis, we found an increasing level of p-Rb in cells treated with miR-224. We therefore concluded that the treatment of cancer cells with miR-224 releases the G1/S checkpoint through the coordinated downregulation of p15, p21, and CCNE1, resulting in increased phosphorylation of Rb and, finally, enhanced cell cycle progression. 

### 3.6. miR-224 Binds to p21 mRNA 3′UTR

**Figure 6:**
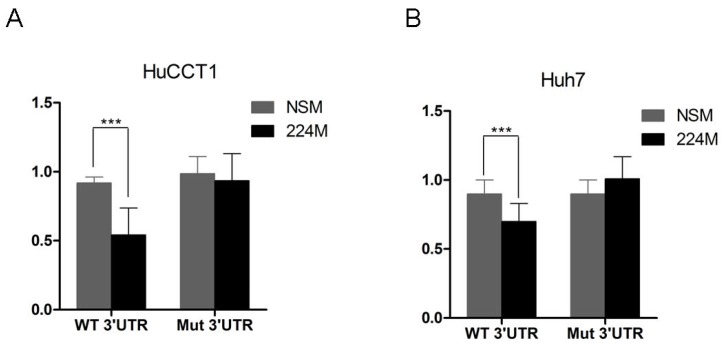
miR-224 directly interacts with the binding site in the 3′UTR of p21 mRNA. Luciferase assay was performed in both HuCCT1 (**a**) and Huh7 (**b**) cells. Y-axis: relative luminescence normalized to the luminescence level in NSM (non-specific mimic) treatment; x-axis: treatment conditions. NSM: non-specific mimic, 224M: miR-224 mimic; WT 3′UTR: correct orientation fragment of p21 3′UTR containing miR-224 binding site; Mut 3′UTR: fragment of p21 3′UTR containing a mutated miR-224 binding site. Shown is the Standard Error of the Mean. miR-224 induces a statistically significant decrease in luminescence (*p*-value < 0.001, Student’s *t*-test) of the forward p21 3′UTR fragment *vs.* NSM in both HuCCT1 and Huh7 cells.

To investigate if miR-224 actions are exerted through direct binding to conserved binding sites in the 3′UTR of the targets identified in the current study, we first performed an *in silico* complementarity search by employing TargetScan [16]. We found that only p21 has a conserved binding site in its 3′UTR, at the position 1524–1530. We next assessed a potential direct interaction between miR-224 and p21 *in vitro* using HuCCT1 and Huh7 cells that were transfected with luciferase reporter plasmids containing the wild-type p21 mRNA 3′UTR as well as miR-224 mimic or NSM. Luciferase activity in the cells co-transfected with miR-224 mimic was down-regulated by ~40% (*p* < 0.001, unpaired Student’s *t*-test) compared with cells co-transfected with NSM. The effect was rescued by using a mutated sequence instead of the p21 wild-type sequence within the 3′UTR (Figure 6a). Similar experiments were conducted in Huh7 cells, where miR-224 decreased the luciferase activity by ~20% (*p* < 0.001, unpaired Student’s *t*-test), an effect that was lost upon mutating the miR-224 binding site (Figure 6b). These data confirmed that miR-224 can bind directly to the 3′UTR of p21 in human liver cancer cells.

## 4. Conclusions

CCA is another malignant tumor in the liver, characterized by high mortality rate and poor prognosis [19]. The pathology of CCA is still unclear and little progress has been made to improve the clinical outcome of CCA patients. The discovery of microRNA deregulation in CCA has added to the knowledge and our understanding of CCA, and it has also presented us with promising novel approaches to diagnose and treat CCA. In the current study, we highlighted one miRNA, miR-224, and investigated its regulatory role in the cell cycle of CCA cells. 

It was observed that miR-224 is upregulated in HCC patients and acts as an oncogene [9]. In the current study, we found miR-224 was upregulated in a large cohort of human CCA *vs.* normal liver tissues, and further study demonstrated miR-224 promotes the growth of cancer cells including cholangiocarcinoma HuCCT1 cells. Others reported miR-224 to be upregulated in the hepatocellular cell line HepG2 and involved in cellular migration and invasion [20], and a recent study reported miR-224 may be a unique biomarker for the early detection of liver malignancies as well as a novel therapeutic target for HCC treatment [21]. Another group was able to demonstrate that increased miR-224 levels in HCC cell lines MHHC97H and MHCC97L targeted HOXD10 and confirmed reduced HOXD10 protein levels in human HCC samples, although the group did not examine miR-224 levels in these samples [22]. We conceived our hypothesis that miR-224 plays a regulatory role in CCA based on the findings above. 

Bioinformatics analysis provides a useful tool to explore how miRNAs target gene function, signaling pathways, and the mRNA interaction networks in physiological as well as pathological conditions [23]. Ingenuity Pathway Analysis (IPA) allowed us to determine the top-ranked diseases and molecular and cellular functions which were regulated by miRNAs [24]. In the current study, based on the mRNA microarray data, IPA was applied to dissect the miR-224 function and we found the top-ranked disease regulated by miR-224 was cancer, and the top three ranked molecular and cellular functions related by miR-224 were “gene expression”, “cell cycle”, and “cell death”. This supported our hypothesis that miR-224 plays important roles in CCA, and further supplied us with the possibility that miR-224 is involved in cell cycle regulation in CCA. To study the precise cell cycle-related molecules impacted by miR-224, the network between miR-224 and mRNA was constructed and it showed that miR-224 appears to regulate several molecules involved in the G1/S checkpoint. The mRNA levels of p15 (INK4), p21(Cip1), and Cyclin E1 (CCNE1) together with their respective protein levels decreased and p-Rb (phospho-Rb) increased in cells following miR-224 stimulation. It was further confirmed by flow cytometry that miR-224 induces G1/S release and promotes the cell cycle process. Protein levels for p15, p21, and CCNE1in the normal cholangiocyte cell line H69 also decreased, although p-Rb levels seemed unaffected. The impact of targeting miR-224 in CCA on normal cholangiocytes remains to be investigated as p-Rb levels did not change upon miR-224 stimulus, but an effect of further downregulation of miR-224 cannot be excluded. 

We did not identify HOXD10 as a target of miR-224 as shown in HCC [22]. It is possible miR-224 targets differ in different tumor types, but it is more likely that the different bioinformatics approaches led to the difference. The IPA pathway analysis led us to look more closely at cell cycle regulatory genes as opposed to HOXD10, as it is associated with cell invasion in HCC. 

miRNAs play important regulatory roles by targeting mRNAs for mRNA degradation or translational repression [25]. In addition to the bioinformatics analysis above, we further verified that miR-224 targeted the p21 mRNA 3′UTR in both HuCCT1 cells and Huh7 cells, using the Dual-Glo Luciferase analysis system. Overexpression of miR-224 decreased p21 protein expression. With the exception of p21, the other molecules including p15, CCNE1, and p-Rb have no direct binding sites for miR-224. As such, this finding invites the conclusion that miR-224 regulates CCA cell growth by suppressing p21 directly, but how it impacts the other molecules remains to be investigated. A recent study of chemoresistance to cisplatin in human lung adenocarcinoma also demonstrates p21 as a direct target of miR-224, confirming our observations [26].

In conclusion, our results suggest that miR-224 enhances G1/S release in CCA via p21 targeting. These findings may shed light on the development of a therapeutic strategy for the treatment of CCA.

## Supplementary Files

Supplementary File 1
