# Supplementary material for: MicroRNA-224 Induces G1/S Checkpoint Release in Liver Cancer
Source: J Clin Med. 2015 Aug 26;4(9):1713–28. doi: 10.3390/jcm4091713 (PMC4600154; doi:10.3390/jcm4091713)
Supplement: Supplementary File 1 [file jcm-04-01713-s001.pdf]

Supplementary Information

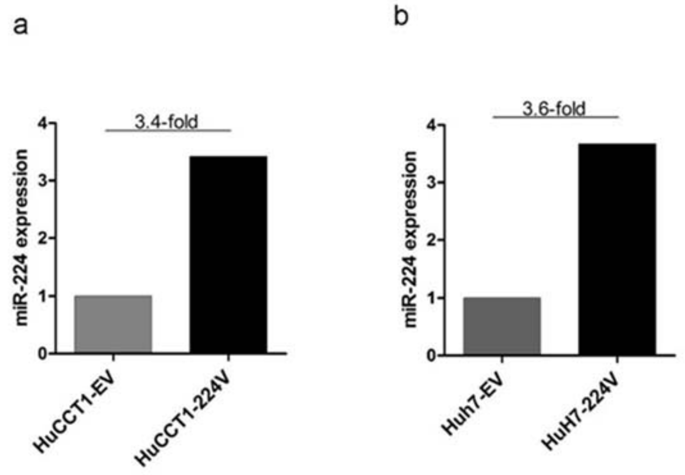

Figure S1. miR-224 is upregulated to physiological levels through retrovirus-mediated delivery. miR-224 is upregulated approximately 3.4- and 3.6-fold when retrovirus-mediated miR-224 delivery is employed in HuCCT1 (**a**) and Huh7 (**b**) cells, respectively. MIEG3 empty virus: EV, MIEG3-miR-224 virus -224V.
